# Establishment of an *in vitro* implantation model using a newly developed mouse endometrial organoid

**DOI:** 10.1242/dev.204461

**Published:** 2025-05-14

**Authors:** Taishi Fujimura, Isao Tamura, Azumi Yoshimura, Toshihide Yoneda, Hitomi Takasaki, Amon Shiroshita, Yuichiro Shirafuta, Shun Sato, Norihiro Sugino

**Affiliations:** ^1^ Department of Obstetrics and Gynecology, Yamaguchi University Graduate School of Medicine, Minamikogushi 1-1-1, Ube 755-8505, Japan; ^2^ Yamaguchi University Graduate School of Medicine, Minamikogushi 1-1-1, Ube 755-8505, Japan

**Keywords:** Implantation, Endometrial organoid, Decidualization, Apical-out

## Abstract

Implantation failure is a major cause of infertility, but its mechanisms remain unclear due to the lack of techniques for constructing organized endometrial structures and recapitulating the implantation process *in vitro*. Endometrial organoids have recently been developed, but they consist of only epithelial cells, and their apical surface faces inward, preventing blastocyst attachment. We developed an apical-out mouse endometrial organoid incorporating epithelial and stromal cells, and examined its ability to recapitulate implantation with mouse blastocysts. Mouse uteri were digested with collagenase and cultured in monolayers. The resulting aggregates were then transferred to low-attachment plates for 3D culture. After 7 days, self-organized aggregates contained E-cadherin-positive epithelial cells outside and vimentin-positive stromal cells inside. Mucin 1 signals were observed on the apical side of epithelial cells, confirming the apical-out orientation. Organoids were stimulated with sex steroid hormones and co-cultured with blastocysts. Time-lapse imaging revealed the four implantation steps: blastocyst attachment, epithelial invagination, entosis and invasion. Invaded cells expressed proliferin while surrounding stromal cells expressed cyclooxygenase 2, indicating trophoblast differentiation and decidualization. This novel organoid closely recapitulates the mouse endometrium and implantation process *in vitro*.

## Teaser

**Highlighted Article:** A novel apical-out endometrial organoid with stromal cells established under ECM-free conditions, enabling the recapitulation and real-time observation of the implantation process in three dimensions by co-culturing with blastocysts.

## INTRODUCTION

Implantation is the process during which an embryo attaches to the uterine epithelium and invades the underlying uterine stroma, followed by the formation of the chorionic structure (Cha et al., 2012). This process is regulated by complex mechanisms, including crosstalk between endometrial cells and the embryo. Implantation failure is a leading cause of infertility (Bashiri et al., 2018). However, the detailed mechanisms underlying human implantation have not been elucidated (Cha et al., 2012), mostly due to the ethical difficulties of analyzing the implantation process *in vivo*. Therefore, there has been much interest in developing a system to reproduce and monitor the implantation process *in vitro*. The process of implantation in mice has been considered to consist of four steps (Fig. S1A): (1) ‘attachment’ of the blastocyst to the endometrial epithelial cells (EECs); (2) invagination of the endometrial epithelium (formation of ‘implantation chamber’); (3) engulfment of EECs by trophoblast cells (‘entosis’); and (4) ‘invasion’ of the blastocyst into the stroma. Previous studies have attempted to reproduce the implantation process *in vitro*. Initially, blastocysts or aggregates of trophoblast cell lines were placed on top of a two-dimensional culture of EECs or endometrial stromal cells (ESCs) (Evans et al., 2020; Lindenberg et al., 1985; Tan et al., 2005). Later, layered three-dimensional (3D) co-cultures of EECs and ESCs were developed to mimic the 3D structure of the endometrium (Bentin-Ley et al., 2000). However, there is still no reliable model that adequately reconstructs the endometrial structure (Li et al., 2022). Therefore, it has been difficult to reproduce the implantation process *in vitro*.

The rapid development of organoid culture technology has enabled the study of epithelial biology in many organs (Haider and Beristain, 2023; Hofer and Lutolf, 2021; Li et al., 2022). Endometrial organoids have been established as a novel *in vitro* 3D culture model of EECs (Boretto et al., 2017; Turco et al., 2017) and are promising models for studying the implantation process. However, endometrial organoids have some limitations as *in vitro* implantation models. First, EECs of endometrial organoid have a basal-out/apical-in polarity (Fig. S1B) (Turco et al., 2017). Since blastocysts access the apical surface of the epithelium during implantation *in vivo*, the organoids cannot recapitulate the blastocyst attachment unless a blastocyst is injected into the central lumen of the organoid, which is technically challenging. Second, the organoids are cultured within an extracellular matrix (ECM) such as Matrigel (Rawlings et al., 2021; Turco et al., 2017). This also prevents the blastocyst from directly contacting the EECs even if they are co-cultured. A third limitation is the lack of ESCs under the epithelial layer (Turco et al., 2017). Therefore, the process of the blastocyst invading the stromal layer cannot be reproduced. In addition, not only EECs, but also ESCs, play important roles in the implantation process (Matsumoto et al., 2018). ESCs undergo dramatic morphological and functional differentiation during implantation, called decidualization, which is crucial for successful implantation (Gellersen et al., 2007; Gellersen and Brosens, 2003, 2014; Tamura et al., 2023a). Therefore, to fully recapitulate implantation *in vitro*, it is essential to generate an endometrial organoid containing both EECs and ESCs without an ECM (Fig. S1B). Very recently, a few endometrial-like 3D structures have been reported that address these limitations (Ahmad et al., 2024; Rawlings et al., 2021; Shibata et al., 2024). However, so far, there are no reports of organoids that have succeeded in overcoming the above three limitations and reproducing the implantation process.

In this study, we have developed a novel apical-out mouse endometrial organoid formed by EECs outside and ESCs inside without embedding in an ECM. By co-culturing the organoids with blastocysts, we have succeeded in recapitulating the mouse implantation process *in vitro*.

## RESULTS

### Development of a novel endometrial organoid

**Fig. 1.**
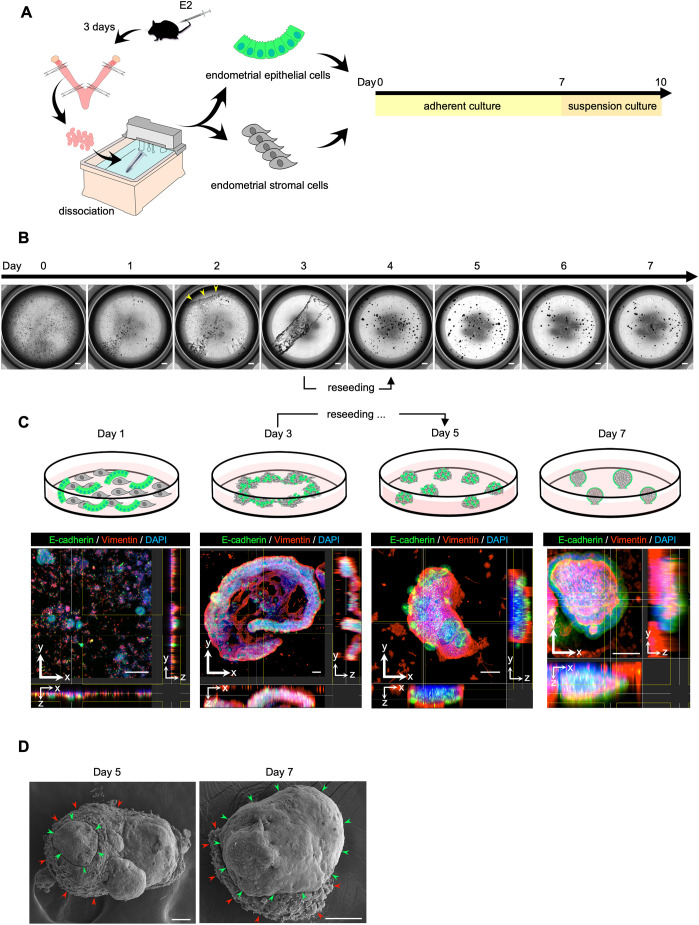
**Aggregate formation under adherent co-culture of EECs and ESCs.** (A) Schematic of the culture procedure. (B) Representative bright-field images of the adherent co-culture of EECs and ESCs. Spontaneous aggregation of EECs and ESCs was observed from day 2 (yellow arrowheads) and formed a large ring-like structure on day 3. They were dissociated into smaller aggregates and reseeded onto the same well (day 4). On day 7, these aggregates developed into a three-dimensional, omega-shaped structure, with only the bottoms attached to the dish. Scale bars: 1000 μm. (C) Schematic and 3D immunostaining images of the aggregates under adherent co-culture. Representative cross-sectional slices of the aggregates in *x-y*, *x-z* and *y-z* planes are shown. Images were taken by confocal microscopy. E-cadherin for EECs; vimentin for ESCs; DAPI for nuclei. Scale bars: 100 μm. The localization of EECs and ESCs is shown in the schematic; EECs are shown in green and ESCs are shown in gray. (D) Representative scanning electron microscopy (SEM) images of the aggregates under adherent co-culture. On day 5, EECs demonstrated by the smooth surface (green arrowheads) partially localized to the outside of the aggregates. On day 7, the aggregates were almost covered by EECs (green arrowheads), except for the site attached to the dish where ESCs remained exposed (red arrowheads). Scale bars: 50 μm.

Mouse EECs and ESCs were simultaneously seeded onto an adherent culture plate with a medium previously established for endometrial organoid culture (Boretto et al., 2017) (Fig. 1A). Spontaneous aggregation of EECs and ESCs was observed from day 2 (Fig. 1B, day 2). Time-lapse imaging revealed that cells aggregated from the periphery of the well towards the center, ultimately forming a large ring-like structure encompassing almost all cells on the dish (Fig. 1B, day 3) ( Movie 1). Immunostaining of the aggregates revealed a disordered arrangement of vimentin-positive ESCs and E-cadherin-positive EECs (Fig. 1C, day 3). We gently dissociated the large aggregate into smaller aggregates by pipetting and reseeded them onto the same well. The reseeded aggregates on day 5 were formed by a striking reorganization of cell types: EECs were preferentially localized to the periphery, while ESCs occupied the inside of the aggregates (Fig. 1C, day 5). On day 7, these aggregates developed into a three-dimensional, omega-shaped structures, with only the bottoms attached to the dish. EECs mostly enveloped the outside and ESCs filled the inside (Fig. 1C, day 7). Scanning electron microscopy (SEM) of the reseeded aggregates showed a smooth surface (indicating EECs) covering most of the outside of the aggregates (Fig. 1D, day 5, green arrowheads). On day 7, the aggregates were mostly covered by EECs (Fig. 1D, day 7, green arrowheads), except for the site attached to the dish, where ESCs remained exposed (Fig. 1D, day 7, red arrowheads). This specific arrangement of EECs and ESCs indicates the aggregates were formed by self-organization, a hallmark of organoid culture (Hofer and Lutolf, 2021). The adherent culture using conventional cell culture medium (DMEM with 10% FBS) instead of the organoid medium did not form aggregates (Fig. S2), indicating that the organoid medium was essential for their formation. Furthermore, the adherent culture of EECs alone using organoid medium, without ESCs, did not form aggregates (Fig. S3), highlighting the crucial role of ESCs in the formation of the aggregate.

**Fig. 2.**
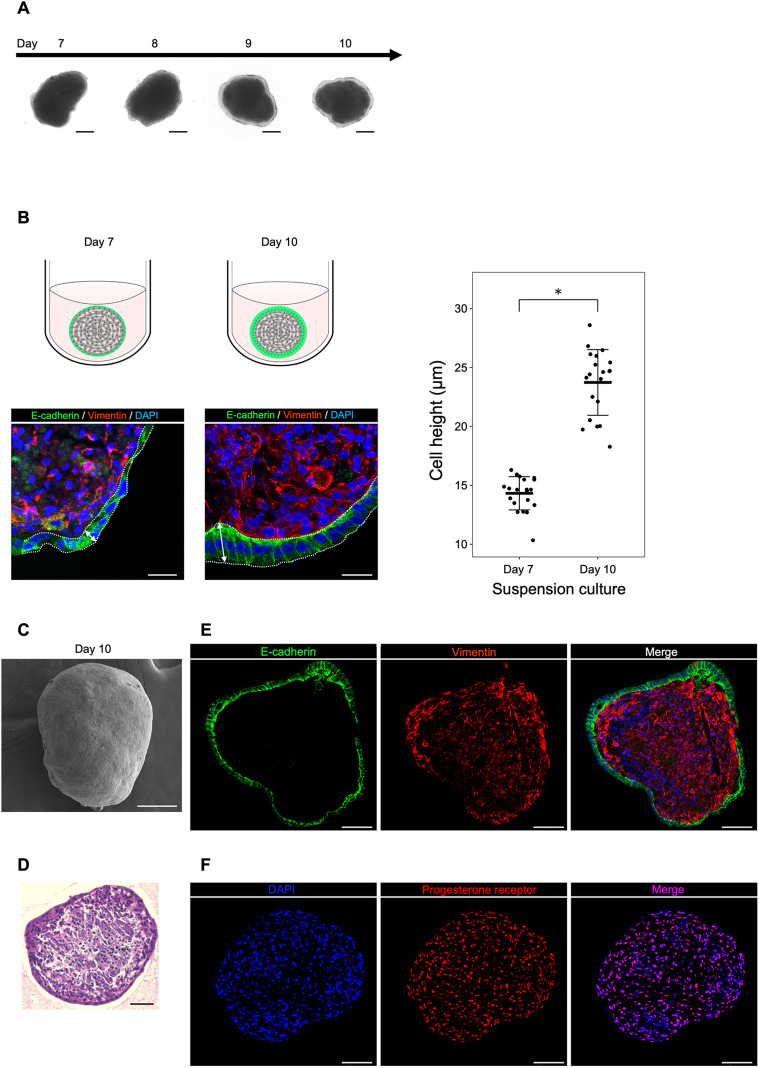
**Suspension culture facilitates the formation of endometrial organoids composed of EECs outside and ESCs inside.** (A) Bright-field images of suspension cultures of aggregates. The picked-up aggregates were transferred to low-attachment U-bottom dishes for suspension culture. During the 3 days of suspension culture, a transparent layer covering the outer surface gradually thickened and eventually enveloped the entire aggregate. Scale bars: 100 μm. (B) Schematic and immunostaining images of the aggregates under suspension culture. (Left) Representative image of the immunostaining for frozen sections of the aggregates are shown: E-cadherin for EECs; vimentin for ESCs; DAPI for nuclei. Scale bars: 20 μm. Arrows indicate the measured height of EECs. (Right) Quantification of the cell height of EECs before (day 7) and after (day 10) suspension culture is shown. Data are mean±s.d. of 15 aggregates in each group. Each data point is indicated as a dot. *P<0.01 (two-sided Student's *t*-test). (C) Representative scanning electron microscopy image of the aggregate after suspension culture. All surfaces of the aggregate were covered with smooth EECs, with no exposed ESCs. Scale bar: 100 μm. (D) A representative HE-staining image for frozen section of the aggregates after suspension culture. Scale bar: 50 μm. (E) Representative immunostaining images for frozen section of the aggregates after suspension culture. E-cadherin for EECs; vimentin for ESCs; DAPI for nuclei. Scale bars: 50 μm. (F) Representative immunostaining images of a progesterone receptor in frozen sections of the aggregates after suspension culture. DAPI indicates nuclei. Scale bars: 50 μm.

The aggregates were picked up and transferred to low-attachment U-bottom dishes for suspension culture. During the first 3 days of suspension culture, a transparent layer covering the outer surface gradually thickened and eventually enveloped the entire aggregate (Fig. 2A). This transparent layer was an epithelial cell layer composed of E-cadherin-positive EECs (Fig. 2B). During this process, squamous EECs became columnar cells with increasing cell height (Fig. 2B). SEM revealed that all surfaces of the aggregate were covered with smooth EECs, with no exposed ESCs observed (Fig. 2C). These results indicated that the suspension culture was essential for the generation of organoids covered with EECs. Histological sections of the aggregates revealed a single outer layer of E-cadherin-positive EECs, and the inner layer was densely filled with vimentin-positive ESCs (Fig. 2D,E). Almost all EECs and ESCs were positive for progesterone receptor (Fig. 2F). Other epithelial markers (EpCAM and CK8) and a mesenchymal marker (PDGFRα) were also expressed in their respective cell types (Fig. S4A). 48.6±5.6% (mean±s.d. of five organoids) of epithelial cells were positive for FOXA2, a marker for glandular epithelial cells (Kelleher et al., 2017) (Fig. S4B). This indicated that the remaining half of EECs consisted of luminal epithelial cells. All of inner layer cells were negative for CD31 (vascular cell maker) and CD45 (immune cell marker), indicating that ESCs were major component of the inner layer (Fig. S5A,B). In addition, an abundant presence of collagen was observed in the inner layer (Fig. S5C), which is consistent with the collagen distribution in the stromal layer of mouse endometrium (Gnecco et al., 2023). The organoid began to disaggregate from day 15, indicating that its viability could not be maintained for an extended period. Furthermore, the organoid could not be passaged. When EECs and ESCs were cultured directly in suspension without undergoing adherent culture, aggregates were formed. However, EECs localized inside the aggregates and did not cover the aggregate surface, whereas ESCs localized outside the aggregates (Fig. S6, day 7). The aggregates began to disaggregate after 10 days of cell culture (Fig. S6, day 10). Taken together, these results demonstrate that it is possible to create endometrial organoids with ESCs inside and EECs outside that do not require embedding in an ECM.

### The novel endometrial organoid exhibits apical-out polarity

**Fig. 3.**
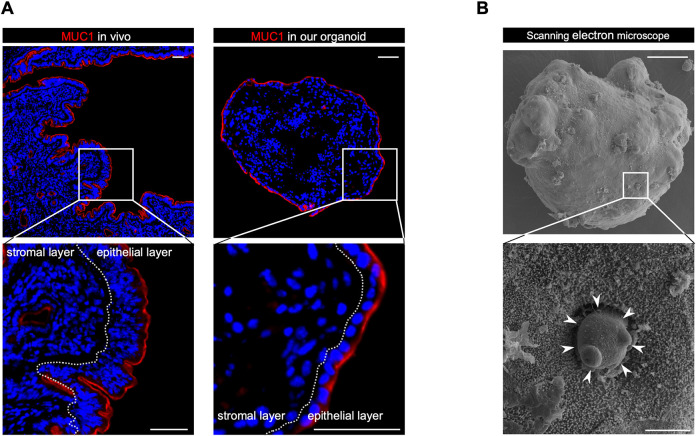
**Novel endometrial organoids exhibit apical-out polarity.** (A) Representative immunostaining images of MUC1 in the mice endometrium (left) and our endometrial organoid (right). The dotted lines indicate the border between the epithelial and stromal layers. DAPI indicates nuclei. Scale bars: 50 μm. (B) Scanning electron microscopy revealed the presence of microvilli, which are characteristic of the apical surface, on the surface of the organoids. Large dome-shaped protrusions were also observed on the surface, which were similar to pinopodes (arrowheads). Scale bars: 50 μm (top); 5 μm (bottom).

Organoid need apical-out polarity in order to recapitulate the implantation process *in vitro* when co-cultured with blastocysts. Mucin 1 (MUC1), a protein highly expressed on the apical side of luminal epithelial cells (Fig. 3A, left) (Brayman et al., 2004), was strongly expressed on the outer surface of EECs (Fig. 3A, right). Furthermore, SEM revealed the presence of microvilli, a characteristic of apical surface (Whitby et al., 2020), on the surface of the organoids (Fig. 3B). Notably, large dome-shaped protrusions were also observed on the surface (Fig. 3B, white arrowheads). These structures are similar to pinopodes, which are well known to be expressed on the surface of luminal epithelial cells of the endometrium (Quinn et al., 2020). These results showed that the organoids possess an apical out-epithelial polarity, which suggests they have the potential to recapitulate *in vitro* implantation when co-cultured with blastocysts.

### Establishment of an *in vitro* implantation model

**Fig. 4.**
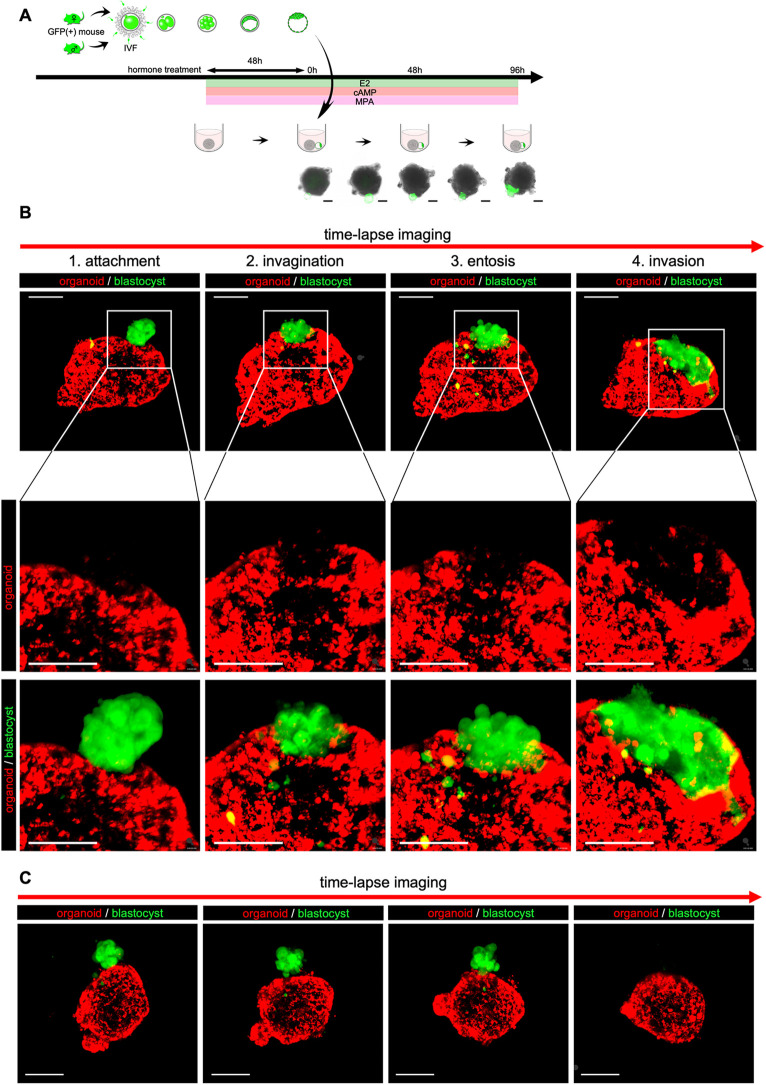
**Time-lapse imaging of an *in vitro* implantation model with a novel endometrial organoid.** (A) Schematic of the *in vitro* implantation model. To induce a receptive state for blastocysts in the organoids, the organoids were treated with E_2_, cAMP and MPA for 48 h. The organoids were then co-cultured with blastocysts obtained from GFP transgenic mice in low-attachment U-bottom dishes. The representative bright-field images of the *in vitro* implantation process are shown. Blastocysts (GFP) attached to the organoids ∼48 h after the initiation of the co-culture. By 96 h, the blastocysts invaded the inside of the organoids. Scale bars: 100 μm. (B) Time-lapse imaging of *in vitro* implantation. After hormone treatment, the organoids were labeled with a red fluorescent dye and co-cultured with blastocysts (green). The process of *in vitro* implantation was monitored using time-lapse imaging from 48 h to 96 h after the initiation of the co-culture. Blastocysts attached to the surface of the organoids (attachment). EECs then invaginated, forming a cavity enveloping the blastocyst (invagination), and EECs were endocytosed by GFP-positive blastocysts (entosis-like cell-in-cell invasion). Finally, blastocysts invaded the ESC layer beneath the EEC layer (invasion). Scale bars: 100 μm. (C) Time-lapse imaging of co-culture of blastocysts (green) and organoids (red) without hormone pretreatment. The organoids without hormone treatment were co-cultured with blastocysts. Blastocyst behavior was monitored using time-lapse imaging from 48 h to 96 h after the initiation of the co-culture. Blastocysts did not attach to the organoid. Scale bars: 100 μm.

**Fig. 5.**
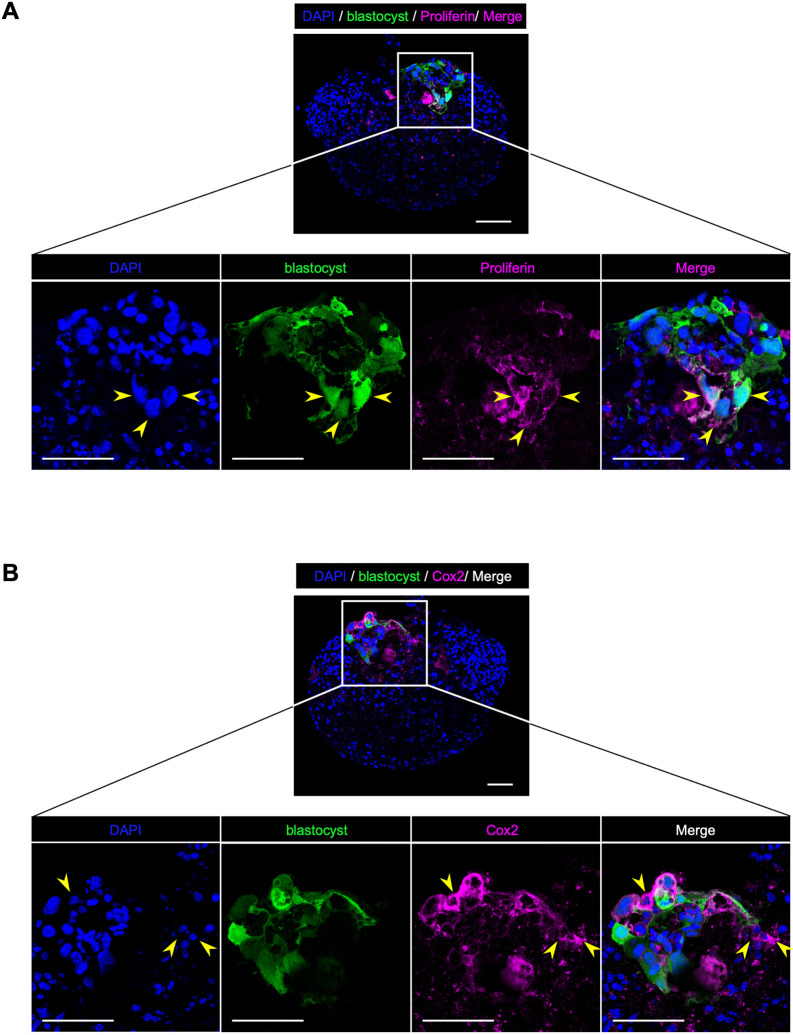
**Implantation responses occurred during *in vitro* implantation.** (A) Representative immunostaining images of proliferin in frozen section of implantation-positive organoid. The nuclei are stained with DAPI. Arrowheads indicate the blastocyst-derived cells that are positive for proliferin with a large nucleus. Scale bars: 50 μm. (B) Representative immunostaining images of Cox2 in frozen sections of implantation-positive organoid. The nuclei were stained with DAPI. Arrowheads indicate the ESCs surrounding the invading blastocyst-derived cells that are positive for Cox2. Scale bars: 50 μm.

Our method to establish an *in vitro* implantation model using the organoids is shown in Fig. 4A. To induce a receptive state for blastocysts in the organoids, the organoids were treated with or without estradiol (E_2_), cAMP and medroxy progesterone acetate (MPA) for 48 h. To examine whether the organoid can respond to the hormones to be in a receptive state for blastocysts, the markers for receptive endometrium were examined. The hormone treatment upregulated FOXO1 (Fig. S7) and downregulated PR (Fig. S8) in the epithelial cells, which are well-known changes on the receptive endometrium (Vasquez et al., 2018). These results showed that our organoid can respond to the hormone treatment to recapitulate the receptive endometrium. However, in contrast to *in vivo* endometrium (Quinn and Casper, 2009), the number of pinopodes was not increased by the hormone treatment (Fig. S9). Blastocysts were obtained from GFP transgenic mice (Fig. S10). Hormone-treated organoids were co-cultured with blastocysts in low-attachment U-bottom dishes. The blastocysts attached to the organoids approximately 48 h after the initiation of the co-culture. By 96 h, the blastocysts invaded the inside of the organoids (Fig. 4A). To monitor this process in real time, the organoids were labeled with a red fluorescent dye prior to co-culture using a live cell tracking assay (Fig. S11). The attachment and subsequent invasion of blastocyst were then monitored using time-lapse imaging from 48 h to 96 h after the initiation of the co-culture (Fig. S12). Initially, blastocysts attached to the surface (EEC layer) of the organoids (Fig. 4B1, attachment). EECs then invaginated into the ESC layer, forming a cavity enveloping the blastocyst (Fig. 4B2 and Fig. S13A, invagination), and EECs were almost obliterated by GFP-positive blastocysts (Fig. 4B3 and Fig. S13B). This phenomenon might reproduce cell-in-cell invasion, known as entosis (Li et al., 2015; White, 2007). Finally, GFP-positive blastocysts invaded the ESC layer beneath the EEC layer (Fig. 4B4, invasion). Notably, blastocysts did not attach to the organoids that had not been pre-treated with hormones (Fig. 4C). Although the entire process took 9 days from the start of 3D culture to the completion of *in vitro* implantation, no apoptotic cells were observed in the organoids cultured for 9 days in 3D (Fig. S14). Reconstruction of the time-lapse images enabled real-time observation of the implantation process in 3D (Movie 2). Furthermore, to verify the effect of hormone treatment on implantation, 26 samples from each of the hormone-treated and untreated groups were evaluated for implantation. To accurately assess whether implantation was achieved, after co-culture with blastocysts, the organoids were cleared with a tissue-clearing reagent (Fig. S15), and whole-mount immunostaining for E-cadherin was performed to visualize the EEC layer. The organoids were then evaluated by whole-organoid imaging at single-cell resolution (Movie 3). Implantation was defined as the invasion of GFP-positive blastocyst-derived cells within the ESC layer under the EECs. The implantation rate in the hormone-treated group was 73.1% (19/26), significantly higher than the 15.3% (4/26) in the untreated group (Fig. S16). We further investigated whether physiological implantation responses occurred during *in vitro* implantation. Blastocyst-derived cells that invaded the ESC layer had large nuclei and were positive for proliferin, a marker for trophoblast giant cells (TGCs) (Fig. 5A, arrowheads). This indicated that placental villous differentiation was induced during *in vitro* implantation. Additionally, ESCs surrounding the invading blastocyst-derived cells were positive for Cox2, a decidualization marker of ESCs. This indicated that decidualization of ESCs was induced during *in vitro* implantation (Fig. 5B). Taken together, these results show that our endometrial organoids can respond to hormones to alter endometrial receptivity, and possess the ability to recapitulate the early implantation process.

## DISCUSSION

Implantation remains poorly understood because it occurs within the concealed uterine environment. The recapitulation of implantation *in vitro* requires the construction of an endometrium-like structure that closely resembles the *in vivo* environment. Recent advances in organoid culture technology have led to the development of endometrial epithelial organoids cultured in Matrigel (Boretto et al., 2017; Sato et al., 2009; Turco et al., 2017). However, due to their apical-in polarity, it was difficult to recapitulate blastocyst attachment to EECs *in vitro*, as observed during *in vivo* implantation. Therefore, the development of apical-out endometrial organoids was necessary for carrying out *in vitro* implantation studies. Our organoid showed apical-out polarity, which was evidenced by the expression of MUC1, microvilli and pinopodes. This made it possible to recapitulate blastocyst attachment to EECs *in vitro*. Another problem of conventional endometrial organoids was their lack of ESCs (Haider and Beristain, 2023). Implantation is a complex process that involves not only the epithelium but also the subsequent invasion of trophoblasts into the decidualized stromal cell layer (Vento-Tormo et al., 2018). Decidualized ESCs acquire a number of cellular functions that are crucial for successful pregnancy (Gellersen et al., 2007; Gellersen and Brosens, 2003, 2014; Tamura et al., 2021a,b, 2023a,b, 2020). In addition, the attachment of the blastocyst to EECs is regulated in a complex manner by the signaling from ESCs to EECs (Matsumoto et al., 2018). These facts emphasize the indispensable role of ESCs for successful implantation. ESCs were incorporated beneath the epithelial layer in our organoid. Importantly, the stromal layer of our organoids is not a single layer, but is composed of dense cell layers, as occurs *in vivo* (Fonseca et al., 2012; Garcia-Alonso et al., 2021; Wood et al., 2007). Another advantage of our organoids is that they did not need to be embedded in ECM, which leaves the epithelial cells exposed. Under the co-culture conditions, the presence of an ECM may prevent direct contact between EECs and blastocysts (O'Connor et al., 2020). Although some recent reports on the construction of endometrial organoids have addressed each of these limitations separately (Ahmad et al., 2024; Rawlings et al., 2021; Shibata et al., 2024), this is the first report of an organoid that has overcome all these limitations.

Our organoids were generated by the spontaneous 3D aggregation of EECs and ESCs that were growing on adherent plates (Fig. 1). This phenomenon was not observed in the culture of EECs alone without ESCs using organoid medium (Fig. S3), highlighting the crucial role of ESCs in cell aggregation. Spontaneous cell aggregation has been reported to occur when tissue-specific cells and mesenchymal stem cells are co-cultured in the adherent culture (Takebe et al., 2015, 2017). Since ESCs have mesenchymal stem cell characteristics (Gargett et al., 2009), it was likely that our organoid was generated by a similar aggregation mechanism. Importantly, the aggregates showed specific spatial localization for EECs and ESCs, with EECs outside and ESCs inside. This indicated that our culture method facilitated the formation of aggregates with cell-specific localization based on the inherent abilities of the cells. This cell-specific arrangement is a hallmark of organoid culture and is referred to as self-organization (Hofer and Lutolf, 2021). Notably, no aggregates were observed when cells were cultured with conventional culture medium instead of organoid medium (DMEM with 10% FBS) (Fig. S2), supporting the idea that self-organization is the driving force for our organoid formation. Cell aggregates formed by self-organization are more similar in structure and function to those found *in vivo* (Brassard and Lutolf, 2019). Therefore, we were able to generate an apical-out structure by positioning the apical surface of EECs away from ESCs, as observed *in vivo*. Interestingly, when the isolated cells were cultured in 3D from the beginning without undergoing adherent culture, the resulting aggregates were not covered with EECs (Fig. S6). This indicated the crucial role of the initial adherent culture step in the self-organization to construct our organoid. It is also interesting to note that the subsequent 3D suspension culture covered the entire organoid with EECs and also transformed the EECs into *in vivo*-like columnar epithelial cells (Fig. 2B). Therefore, this suspension culture was also an essential step to facilitate a more *in vivo*-like organoid. Taken together, our findings represent a novel approach for organoid generation that takes advantage of epithelial and/or stromal self-organization under adherent and/or suspension culture conditions.

Our organoids replicated some of the major characteristics of implantation. Not only the attachment of blastocysts to the organoid, but also the formation of an implantation chamber-like structure (Fukui et al., 2021; Yuan et al., 2018) was observed. As the invasion progressed into the stromal cell layer, the nuclei of the invading cells enlarged and differentiated into proliferin-positive TGCs (Yamaguchi et al., 1994; Yang et al., 2017). Importantly, ESCs surrounding the invading trophoblast showed a decidual response, similar to the *in vivo* situation (Aikawa et al., 2022; Lim et al., 1997). During implantation, the stromal layer becomes dense with decidualized ESCs, which play an important role in trophoblast invasion by interacting with trophoblasts (Gellersen et al., 2007). Therefore, it is essential to reproduce a dense stromal cell layer in organoids. Two previous studies have reported endometrial organoids containing both ESCs and EECs embedded in an ECM. However, in contrast to our organoids, these organoids did not reproduce a dense stromal cell layer. This is likely due to the reduced cell density caused by the presence of an ECM (Rawlings et al., 2021; Shibata et al., 2024). Our organoids were formed by cell self-aggregation without the use of an ECM, which facilitated close cell-to-cell contact and reproduced a dense stromal layer. This, in turn, allowed the study of implantation phenomena within the stromal cell layer.

Importantly, the hormone-treated organoids showed a significantly higher implantation rate than the untreated organoids. This is convincing because the hormone treatment could recapitulate the expression pattern of receptive markers, including FOXO1 and PR. Shibata et al. recently reported an *in vitro* implantation model in which an endometrial organoid was co-cultured with blastoids, which are blastocyst models derived from human embryonic stem cells (Shibata et al., 2024; Yu et al., 2021). However, in contrast to what we observed, hormone stimulation did not affect the rate of blastocyst attachment to the organoids. Their organoids were larger than ours (about 1500 µm compared to 400 µm for our organoids) and floated near the bottom of the V-shaped well, but not near the bottom of a U-shaped well. Therefore, the organoids might occupy almost the entire plane of the well. Under these conditions, the added blastoids would sink and be forced to gravitationally attach to organoids. Because blastocyst attachment to the endometrium is thought to be independent of gravity (Chen et al., 2013), these different conditions from *in vivo* might obscure hormone responsiveness in their model. Therefore, an advantage of our organoids is their ability to recapitulate hormone responsiveness. Furthermore, it should be noted that blastocyst attachment to the organoid (without invasion) was observed at a high frequency (11 out of 26 samples, 42.3%, Fig. S16A) even in the non-hormone-treated organoids. This is not surprising, as a previous report has shown that blastocyst attachment occurs in the uterus of mice that are not treated with estrogen and progesterone, whereas blastocyst invasion into the stromal layer requires hormone treatment (Grant et al., 1975). These findings further support the notion that our *in vitro* implantation model recapitulates the implantation process observed in mice.

One of the limitations of our model is that we did not observe differentiation of the inner cell mass (ICM) component. Recent advances have made it possible to induce differentiation of the ICM up to the post-implantation stage by using culture media specifically designed to maintain the ICM component (Oldak et al., 2023). However, because *in vitro* implantation models require organoids and embryonic components to be cultured in the same well, a major challenge is to develop specific media that support both components simultaneously. Another point is that hormone treatment did not increase the number of pinopodes, which did not fully recapitulate the *in vivo* situation (Quinn and Casper, 2009; Quinn et al., 2020). Further refinement of the organoid generation process is needed to better mimic the *in vivo* endometrium. During *in vitro* implantation, EECs were nearly obliterated by GFP-positive blastocysts. We consider that this phenomenon reproduces cell-in-cell invasion, known as entosis (Li et al., 2015; White, 2007). However, further detailed examination is required to confirm entosis by demonstrating the engulfment of EECs by blastocyst-derived cells. Finally, the organoid could not be passaged. Therefore, our organoid does not contribute to reducing animal use in research. Establishing a passagable organoid in future studies is anticipated.

### Conclusions

We have developed a novel ECM-free organoid formed by apical-out EECs and ESCs. Notably, the organoid generation method developed in this study is a new approach that has not been reported in any other tissue. This organoid enabled the recapitulation of the mouse implantation process. We expect that organoids generated from human endometrial cells will further elucidate the mechanisms underlying human implantation and lead to the development of novel therapeutic strategies for implantation failure.

## MATERIALS AND METHODS

### Animal work

C57/BL6N and C57BL/6-Tg (CAG-EGFP) mice were originally purchased from Japan SLC. All mouse work was carried out under the committee for ethics on animal experiments at Yamaguchi University Graduate School of Medicine. All mice were maintained in the Yamaguchi University animal housing facility at constant temperature, humidity and day/night cycle, with access to water and food without restriction.

### Organoid culture from mouse endometrium

The protocol to generate the endometrial organoid was primarily based on the previously reported method (Boretto et al., 2017), with minor modifications as described below. Briefly, to induce an estrous state in the endometrium, two C57/BL6N mice at 8 weeks of age were subcutaneously injected with an estradiol valerate:progynon depot (0.01 mg/g of the body weight; Fuji Pharmaceutical, 872473) 3 days before being euthanized (Wang et al., 2014). Uterine horns were then dissected, opened, extensively rinsed with ice-cold PBS and minced into 2 mm fragments. The tissues were incubated with 1 mg/ml collagenase (Sigma-Aldrich, C5138) and 0.1 mg/ml DNase I (Sigma-Aldrich, 10104159001) in Ca^2+^/Mg^2+^-free PBS (FUJIFILM, 166-23555) for 30 min at 37°C water bath with gentle shaking at 100 bpm. After adding ice-cold PBS to a final volume of 15 ml, the isolation of epithelial fractions was performed as previously described (Boretto et al., 2017; Sato et al., 2011). Tissue fragments were mechanically dissociated by pipetting up and down at least 10 times and then allowed to settle down under normal gravity for 1 min. The supernatant was collected as it contains the epithelial fraction (Sato et al., 2011). This process was repeated twice. After centrifuging the supernatant at 200 ***g*** for 3 min at 4°C, the pellet was resuspended in basal medium [advanced DMEM (Thermo Fisher Scientific, 12634010) with Glutamax (0.2 mM; Thermo Fisher Scientific, 35050061), HEPES (1 mM; Thermo Fisher Scientific, 15630080)] and centrifuged twice. Finally, the pellet was resuspended with an expansion medium (Boretto et al., 2017) [advanced DMEM supplemented with penicillin/streptomycin (1%; FUJIFILM, 161-23181), Glutamax (2 mM), B27 (2%; Thermo Fisher Scientific, 17504044), N2 (1%; Thermo Fisher Scientific, 17502048), insulin-transferrin-selenium (ITS, 1%; Thermo Fisher Scientific, 41400-045), HEPES (10 mM), nicotinamide (1 mM; Thermo Fisher Scientific, N0636), EGF (50 ng ml^−1^; Thermo Fisher Scientific, PMG8043), FGF-Basic (50 ng ml^−1^; FUJIFILM, 06205181), Noggin (100 ng ml^−1^; Thermo Fisher Scientific, 120-10C), the TGFβ/Alk inhibitor A83-01 (0.5 μM; Thermo Fisher Scientific, 2939/10), R-spondin (200 ng ml^−1^; Thermo Fisher Scientific, 51996611), WNT3A (200 ng ml^−1^; Thermo Fisher Scientific:Cat#315-20)] with Y27632 (10 μm; MedChemExpress, HY-10583) up to 3 ml and kept on ice as the epithelial fraction until the further processing with stromal fraction. Although previous reports used FGF-10 for the expansion of endometrial epithelial organoids (Boretto et al., 2017; Turco et al., 2017), FGF-basic was used instead, as it is the preferred FGF for promoting fibroblast proliferation (Imaizumi et al., 1996). The remaining pellet, from which the supernatant with the epithelial fraction had been removed, was further digested with 1 mg/ml collagenase and 0.1 mg/ml DNase I for 50 min, and was filtered through a 70 μm cell strainer (Falcon, 352350) followed by a centrifuge at 500 ***g*** for 5 min at 4°C. The pellet, designated as stromal fraction, was resuspended in basal medium and washed twice. The preprepared epithelial fractions were added to the stromal fraction and gently mixed. The mixed tissue was seeded onto a 12-well culture plate (Falcon, 353043) with 1 ml of expansion medium without any coating. Cells were incubated at 37°C in a 5% CO_2_ incubator, and the expansion medium was changed on day 1, day 3 and day 5. On day 1, the dishes were washed twice with a basal medium to remove non-adherent cells and then replaced with an expansion medium. On day 2, the epithelial and stromal cells started to aggregate naturally from the edge of the dish towards the center. This process was monitored with a time-lapse imaging using Confocal Quantitative Image Cytometer (CQ-1; Yokogawa). When the aggregates were shaped in a ring-like structure on day 3, they were collected with a 1000 μl pipette tip into a 1.5 ml tube, and disaggregated by pipetting using a 200 μl pipette with the tip slightly trimmed. The small aggregates were reseeded into the same culture dish. On day 7, every aggregate was picked up separately and cultured in a 96-well low-attachment U-bottom dish (Sumitomo Bakelite, MS-9096U) with expansion medium. The medium was changed every other day. After 3 days of suspension culture (on day 10), organoids were used for subsequent experiments.

### Immunofluorescence staining

Immunofluorescence staining of cells under adherent cultures and whole organoids was performed after tissue and cell clearing using CUBIC reagent with the manufacturer's protocol (Susaki et al., 2014; Tainaka et al., 2018). Briefly, the medium was removed from each tissue, followed by the addition of 4% paraformaldehyde and fixation for 24 h at 4°C. Tissues were pretreated with CUBIC-L (Tokyo Chemical Industry, T3740) for 2 days at 37°C and then thoroughly washed with PBS. Subsequently, nuclei were stained with DAPI using the CUBIC-HVTM1 3D nuclear staining kit (Tokyo Chemical Industry, C3709). Tissues were incubated with primary antibodies (Table S1) for 2 days at room temperature with gentle shaking, then washed with PBS three times for 2 h each and incubated with secondary antibodies (Table S1) for 1 day at room temperature with gentle shaking. Post-fixation was performed with 1% formaldehyde, followed by washing with PBS. Tissues were incubated in 50% CUBIC-R+ (Tokyo Chemical Industry, T3983) at 25°C overnight, followed by incubation in CUBIC-R+ for 1 day with gentle shaking. Images were acquired using either CQ1 or confocal microscopy (Stellaris 8; Leica). 3D images were reconstructed by Stellaris 8 or Imaris (Oxford Instruments). Other immunostaining was performed on frozen sections, unless otherwise noted. Fresh tissue samples were fixed in 4% paraformaldehyde for 16 h and washed with PBS. Tissues were then placed in 15% sucrose in PBS for 16 h, followed by 30% sucrose in PBS for 24 h. They were embedded in Optimal Cutting Temperature (OCT) compound (Sakura, 45833). Frozen sections (5 μm) were cut using a cryostat (Leica) and mounted on glass slides. The slides were washed three times with PBS. Sections were permeabilized with 0.3% Triton X-100 (FUJIFILM, A16046) in PBS for 15 min at room temperature, followed by blocking with 5% normal goat serum for 1 h at room temperature to block non-specific reactions. They were incubated with primary antibodies (Table S1) overnight at 4°C. After washing with PBS, they were incubated with appropriate fluorophore-conjugated secondary antibodies (Table S1) for 1 h at room temperature. Nuclei were counterstained with 4′,6-diamidino-2-phenylindole (DAPI) for 15 min at room temperature. Images were acquired using either a BZ-X800 or Stellaris 8.

### TUNEL staining

The apoptosis was examined by TUNEL staining using an In Situ Apoptosis Detection Kit (Takara, MK500) according to the manufacturer's instructions (Sakai et al., 2025). Organoids cultured in 3D for 9 days were used in the analysis because this is the same time period from the start of 3D culture to the end of *in vitro* implantation. Slices of rat mammary glands included in the kit were used as a positive control of apoptotic cells.

### Collagen fiber staining

Organoids cultured in 3D for 3 days (Day 10) were used for the analysis. Collagen fibers were visualized using a Picrosirius Red Stain Kit (Polysciences, 24901) according to the manufacturer's instructions (Umehara et al., 2020).

### Endometrial epithelial cell height analysis

The organoids on day 7 (before suspension culture) and on day 10 (after suspension culture), were frozen, sectioned and subjected to immunofluorescence staining. The maximum diameter of E-cadherin-positive cells was measured in arbitrary sections and defined as cell height. Fifteen samples were measured for each group, and the data were presented as dot plots. Statistical significance was determined using an unpaired *t*-test; results were considered significant if *P*<0.01.

### Scanning electron microscopy

Organoids were fixed with 2% glutaraldehyde and 2% formaldehyde in a buffer [100 mm NaCl, 30 mm HEPES and 2 mm CaCl_2_ (pH 7.4)] for 2 h at room temperature and postfixed with 1% osmium tetroxide in sodium cacodylate buffer [100 mM sodium cacodylate and 2 mm CaCl_2_ (pH 7.0)] for 2 h at 4°C. Osmicated samples were then dehydrated with graded series of ethanol and infiltrated with t-butanol (Wako, 028-03386; Sigma, 471712). All samples were freeze-dried in a vacuum chamber at 0-5°C (VFD-21S; Vacuum Device). Dried samples were coated with 5-nm osmium in an osmium plasma coater (Neoc-ST; Meiwa Fosis). Scanning electron images were acquired using a field-emission scanning electron microscope (Quanta3D FEG, Thermo Fisher Scientific). To examine whether hormone treatment affects the expression of pinopode, organoids were treated with or without estradiol (E_2_, 10^−8^ M), 3′,5'-cyclic adenosine monophosphate (cAMP, 0.5 mM) and medroxyprogesterone acetate (MPA, 10^−6^ M) for 48 h. Surface images of three organoids in each group were then obtained using scanning electron microscopy, and the number of pinopodes was counted.

### Hematoxylin and Eosin staining

Hematoxylin and Eosin (H&E) staining was performed on frozen sections to examine morphological structures, as reported previously (Takagi et al., 2022; Tamura et al., 2014, 2011).

### *In vitro* fertilization

The *in vitro* fertilization (IVF) protocol was based on previously described methods (Tomikawa et al., 2022). Briefly, inbred 2- or 3-week-old (21-28 days old) female C57BL/6-Tg (CAG-EGFP) mice were intraperitoneally injected with 7.5 IU of pregnant mare serum gonadotropin (PMSG) (Aska Animal Health) to stimulate follicular growth, followed by 7.5 IU of human chorionic gonadotropin (hCG) (Aska Animal Health) 48 h later to induce ovulation. Thirteen hours post-hCG injection, females were euthanized and oviducts were removed. Cumulus-oocyte complexes (COCs) were isolated into human tubal fluid (HTF) (Kyudo) medium droplets. Epididymal sperm were collected from inbred C57BL/6-Tg (CAG-EGFP) males (aged 63-70 days) into HTF droplets. Sperm concentration was adjusted to 2×10^2^ sperm/μl, and insemination was performed by adding the sperm to HTF droplets containing COCs. Two hours post-insemination, oocytes were washed ten times in fresh Potassium Simplex Optimized Medium (KSOM) (Kyudo). Six hours post-insemination, only fertilized oocytes with two visible pronuclei were transferred to culture dishes containing KSOM droplets and cultured until day 5. On the morning of day 5, embryos were categorized as hatched or expanded blastocysts. Expanded blastocysts were hatched using acidic Tyrode's solution (Sigma-Aldrich, T1788) (Fan et al., 2022). Hatched blastocysts positive for GFP expression were identified using fluorescence microscopy.

### *In vitro* implantation with our organoid and blastocysts

Organoids were treated with or without E_2_ (10^−8^ M), cAMP (0.5 mM) and MPA (10^−6^ M) for 48 h prior to co-culture with blastocysts. Hatched blastocysts were co-cultured with organoids in 96-well low-attachment U-bottom dishes and imaged daily using an inverted fluorescence microscope (BZ-X800) to monitor blastocyst-organoid attachment and invasion. To visualize *in vitro* implantation in detail, organoids were incubated with 1× Track It Red (Cell Explorer Live Cell Tracking Kit Red Fluorescence; AAT Bioquest, 22623) in culture medium for 20 min at 37°C in a CO_2_ incubator. After washing five times with basal medium, one blastocyst was added to one well of 96-well U-bottom dishes to co-culture with the labeled organoid at 37°C in a CO_2_ incubator. The expansion medium, with or without hormone treatment, was gently replaced every other day. Time-lapse imaging was initiated 48 h after co-culture using CQ-1. Images were acquired every 20 min at 4 μm intervals from the bottom of the dish to a height of 150 μm. The acquired TIFF data were reconstructed into 3D time-lapse images using Imaris software.

### Statistics and reproducibility

Statistical analysis was performed using R (version 4.4.0). All of the statistical methods are described in the figure legends. A two-sided Student's *t*-test was used for Fig. 2B and Fig. S7. Fisher's exact test was used for Fig. S14B to evaluate the differences in implantation rate. Differences were considered significant at *P* <0.01.

## Supplementary Material

10.1242/develop.204461_sup1 Supplementary information
