# Supplementary material for: Establishment of an in vitro implantation model using a newly developed mouse endometrial organoid
Source: Development. 2025 May 14;152(9):dev204461. doi: 10.1242/dev.204461 (PMC12091871; doi:10.1242/dev.204461)
Supplement: Supplementary information [file develop-152-204461-s1.pdf]

Supplementary Figure 1

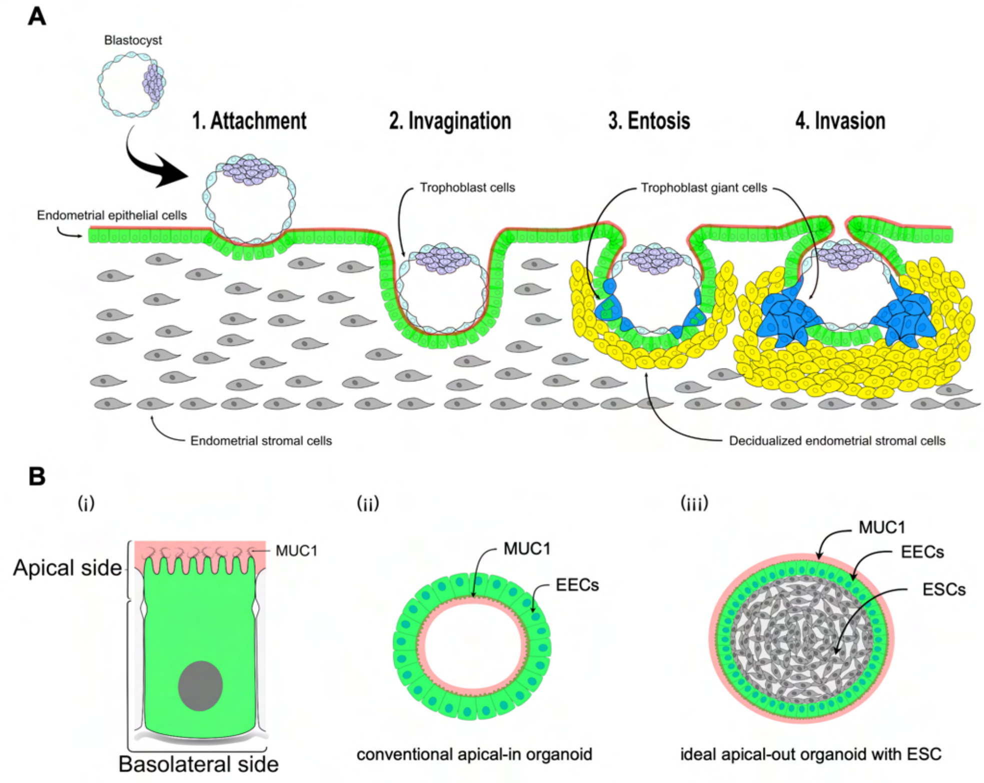

Fig. S1. Scheme of the implantation process and ideal organoid

(A) Scheme of the implantation process in mice.

(B) Comparison of conventional and ideal organoids for in vitro implantation. (¡) Mucin-1 (MUC1) is a protein expressed on the apical side of Endometrial luminal epithelial cells. (¡¡) Conventional endometrial organoid has a basal-out/apical-in polarity and lacks ESCs. (¡¡¡) Ideal organoid for in vitro implantation model is an organoid formed by ESCs and EECs with an apical out epithelial polarity.

Supplementary Figure 2

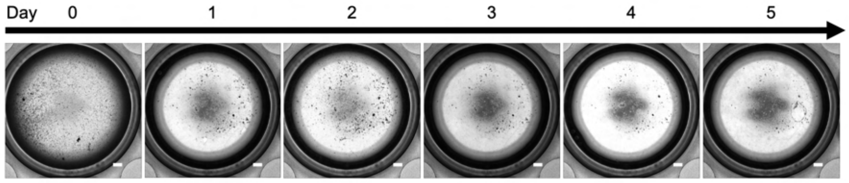

Fig. S2. Representative bright field images of the adherent coculture of EECs and ESCs with conventional cell culture medium (DMEM with 10% FBS). Spontaneous aggregations of EECs and ESCs were not observed. Scale bars, 1000 µm.

Development • Supplementary information

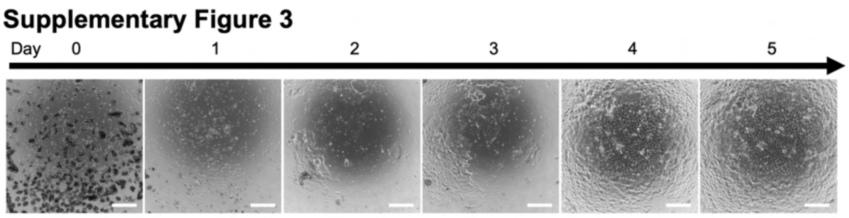

Fig. S3. Representative bright field images of the adherent culture of EECs without ESCs. Spontaneous aggregations of EECs were not observed. Scale bars, 500 µm.

Development • Supplementary information

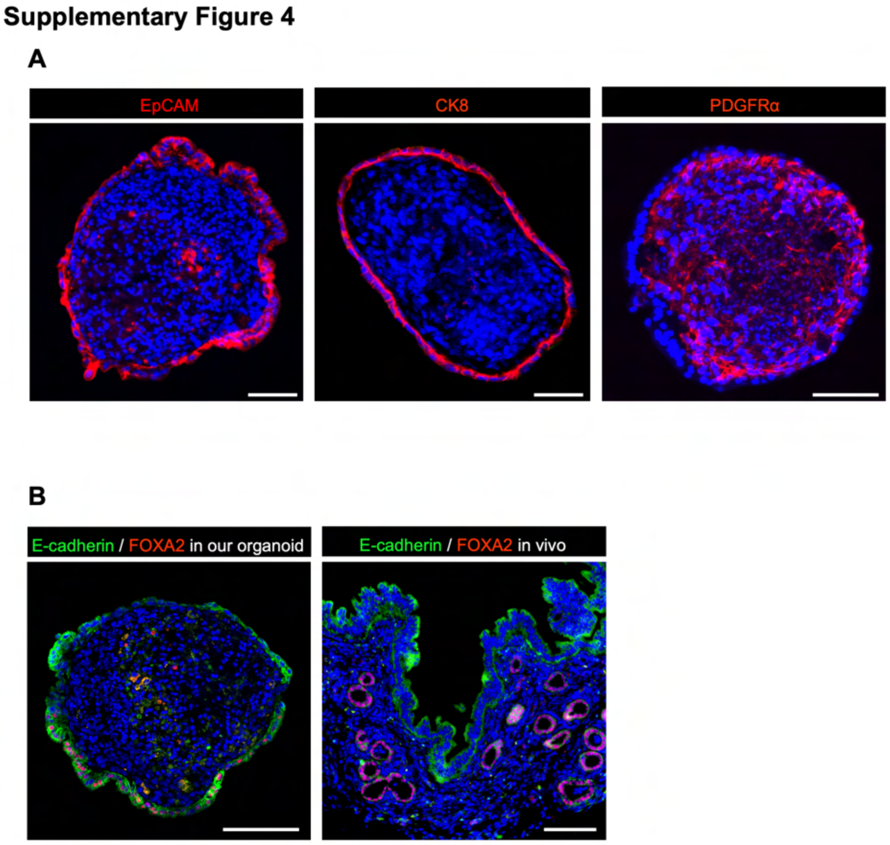

Fig. S4. Representative immunostaining images of the markers for EECs and ESCs.

A. EpCAM and CK8 for EECs; PDGFRα for ESCs; DAPI for nuclei. Scale bars, 100 µm.

B. FOXA2 for glandular epithelial cells (left). Mouse endometrium was used as a positive control (right). E-cadherin for EECs; DAPI for nuclei. Scale bars, 100 µm. The number of FOXA2-positive cells in EECs on a single slice was counted, and the percentage of FOXA2-positive cells in EECs was determined. 48.6±5.6% (mean ± SD of five organoids) of epithelial cells were positive for FOXA2, indicating that the remaining half of EECs consisted of luminal epithelial cells.

Development • Supplementary information

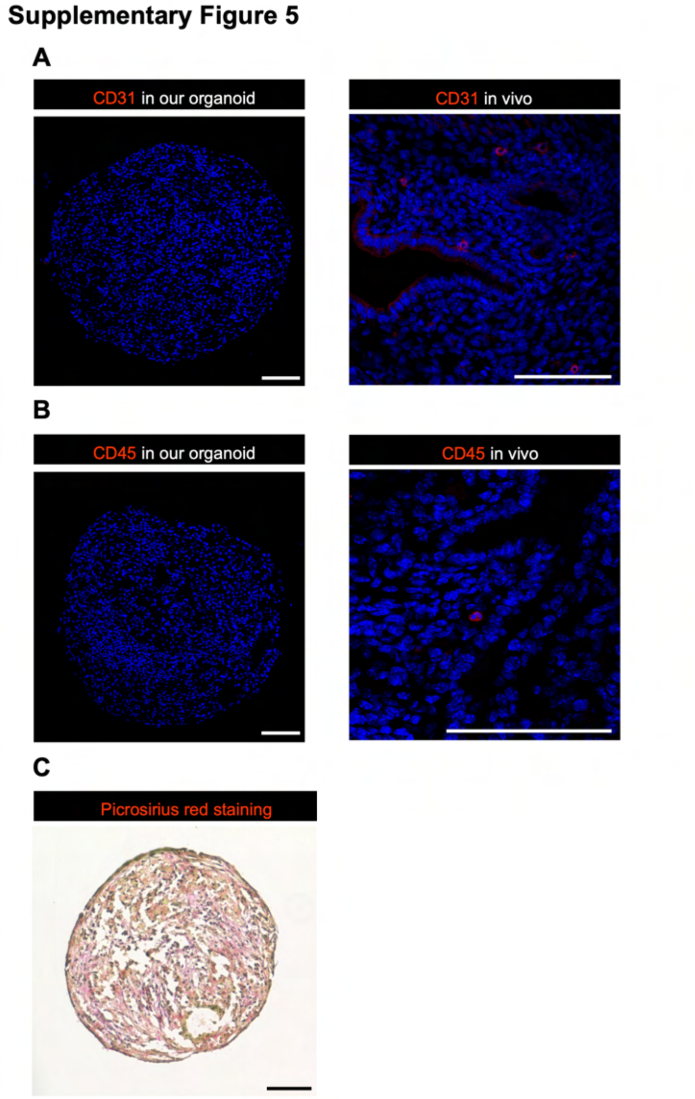

Fig. S5. Representative images of the components of the inner layer.

A, B. Representative immunostaining image of CD31 (A) for vascular cells and CD45 (B) for immune cells. Endometrium from pregnant mice were used as positive controls . No CD31- or CD45-positive cells were detected in the inner layer of the organoid. Scale bar = 100 µm.

C. Representative image of picrosirius red staining of the organoid. Collagen fibers and cellular components are stained pink. Scale bar = 100 µm.

Development • Supplementary information

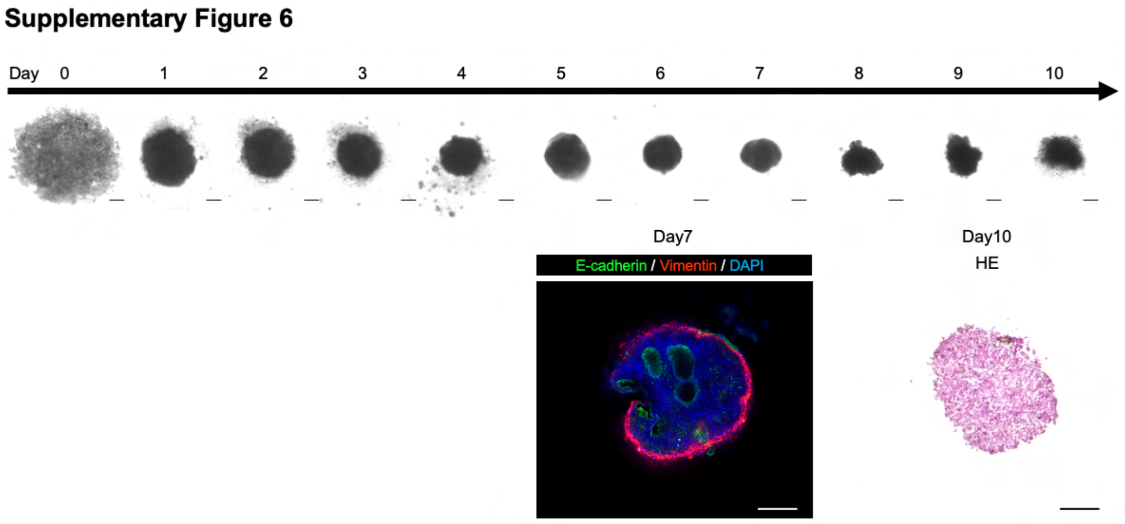

Fig. S6. Suspension coculture of EECs and ESCs without undergoing adherent culture.

Representative bright field images of suspension coculture and whole-mount immunostaining images of the aggregates on day 7 are shown. E-cadherin for EECs; Vimentin for ESCs; DAPI for nuclei. EECs localized inside the aggregates and did not cover the aggregate surface, whereas ESCs localized outside the aggregates. These aggregates were not maintained, as they began to disaggregate after 10 days of suspension culture. A representative image of HE-staining on day 10 is shown. Cells were disaggregated. Scale bars, 100 µm.

Supplementary Figure 7

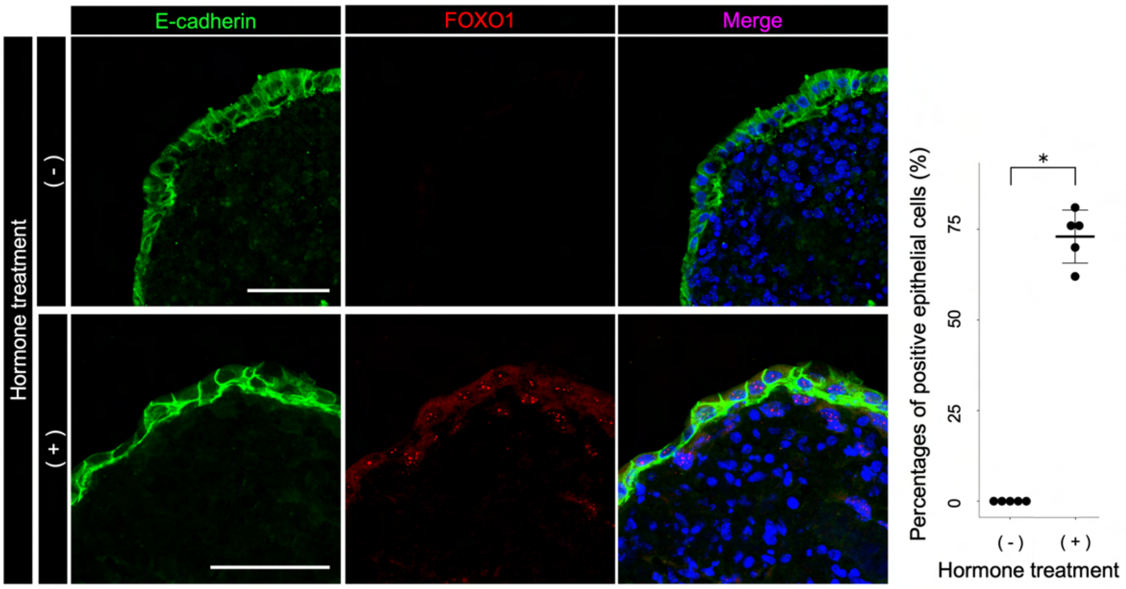

Fig. S7. Effects of hormone treatment on endometrial receptivity markers. Representative immunostaining images of FOXO1 in EECs of the organoid with or without hormone treatment. E-cadherin for EECs; DAPI for nuclei. Scale bars, 50 µm. The number of FOXO1-positive cells in EECs on a single slice was counted, and the percentage of FOXO1-positive cells in EECs was determined. Data are presented as the mean ± SD of five organoids with or without hormone treatment, with each data point indicated as a dot. *, P<0.01 (t-test).

Development • Supplementary information

Supplementary Figure 8

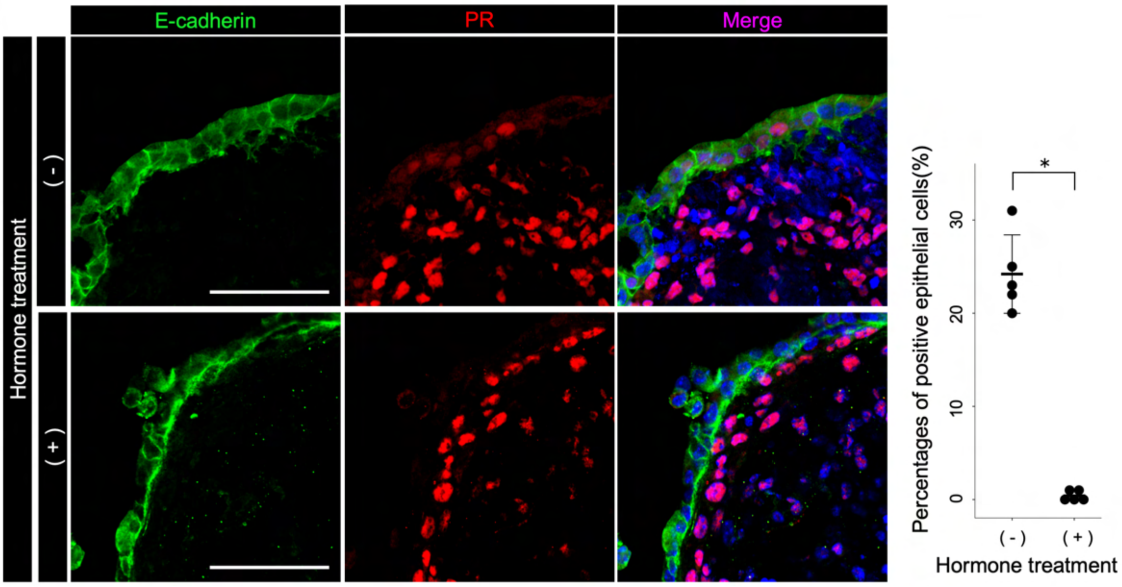

Fig. S8. Effects of hormone treatment on endometrial receptivity markers.

Representative immunostaining images of PR in EECs of the organoid with or without hormone treatment. E-cadherin for EECs; DAPI for nuclei. Scale bars, 50 µm. The number of PR-positive cells in EECs on a single slice was counted, and the percentage of PR-positive cells in EECs was determined. Data are presented as the mean ± SD of five organoids with or without hormone treatment, with each data point Representaindicated as a dot. *, P<0.01 (t-test).

Development • Supplementary information

Supplementary Figure 9

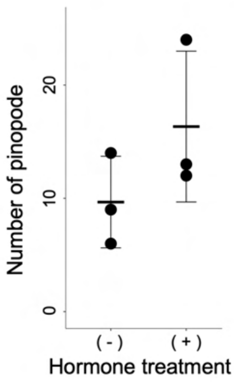

Fig. S9. Effects of hormone treatment on endometrial receptivity markers.

Number of pinopodes on the surface of organoids. Surface images of organoids with or without hormone treatment were obtained using SEM. The number of pinopodes was compared between organoids with and without hormone treatment. Data are presented as the mean ± SD of three organoids with or without hormone treatment, with each data point indicated as a dot.

Supplementary Figure 10

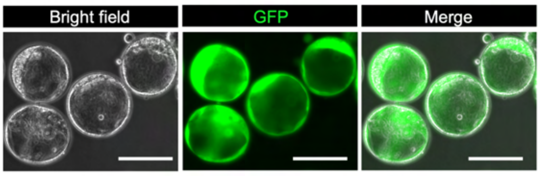

Fig. S10. Representative images of blastocysts obtained from GFP transgenic mice.

Scale bars, 100 μm.

Supplementary Figure 11

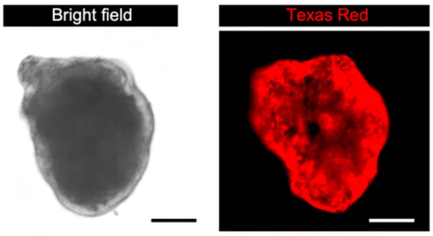

Fig. S11. Representative images of the organoid labeled with a red fluorescent dye using a live cell tracking assay.

Scale bars, 100 μm.

Development • Supplementary information

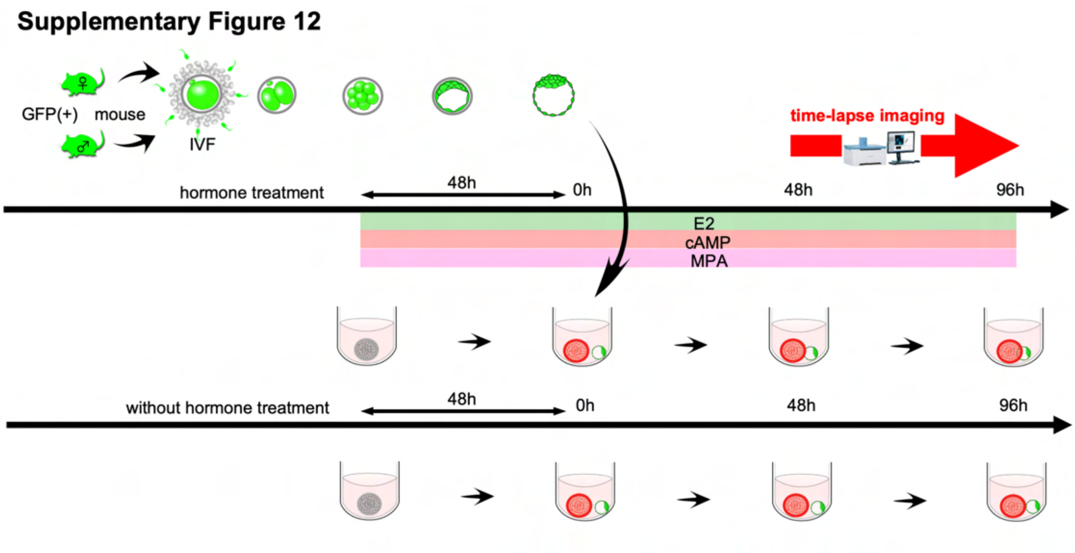

Fig. S12. Scheme of time-lapse imaging of in vitro implantation.

The organoids were pretreated with or without hormones for 48 hours. Then, the organoids labeled with a red fluorescent were cocultured with blastocysts. The process of in vitro implantation was monitored by time-lapse imaging from 48 hours to 96 hours after the initiation of the co-culture.

Development • Supplementary information

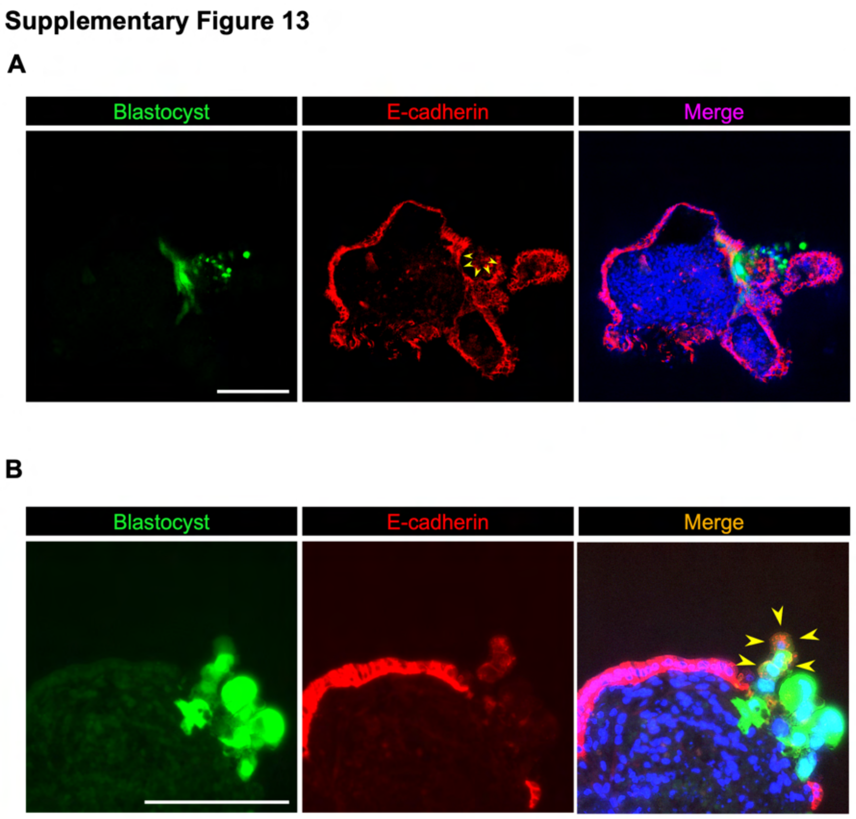

Fig. S13. Representative immunostaining images showing the process of invagination and entosis-like phenomenon.

A. Representative confocal image showing the process of invagination. EECs (red) invaginated, forming a cavity enveloping the blastocyst (green).

B. Representative immunostaining image showing the process of **entosis-like phenomenon**. Frozen sections of the co-cultured organoids were stained with E-cadherin to visualize the EEC layer. The nuclei were stained with DAPI. Arrowheads indicate GFP-positive blastocyst-derived cells., colocalized with EECs, indicating the engulfment of EECs by trophoblast cells. Scale bars, 100 µm.

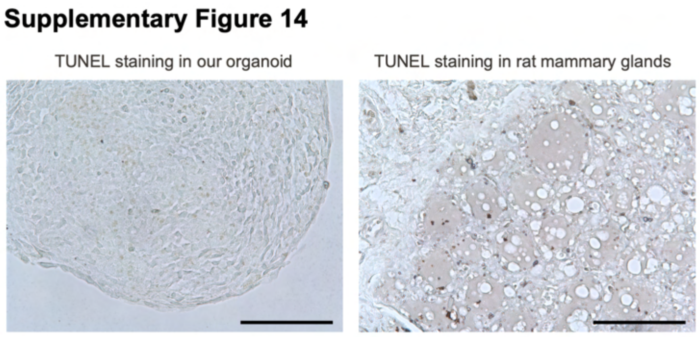

Fig. S14. Detection of apoptotic cells in the organoid by TUNEL staining.

The organoids cultured in 3D for 9 days were analyzed (left) because this is the same time period from the start of 3D culture to the end of in vitro implantation. Slices of rat mammary glands included in the kit were used as a positive control of apoptotic cells (right). Scale bar = 100 µm.

Development • Supplementary information

Supplementary Figure 15

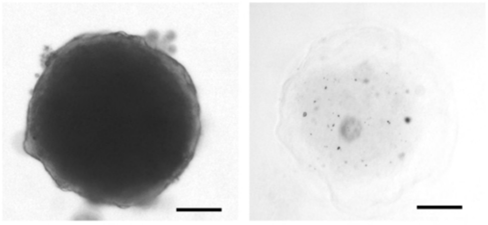

Fig. S15. Representative images of the organoid cleared with a tissue-clearing reagent. Scale bars, 100 µm.

Development • Supplementary information

Supplementary Figure 16

A

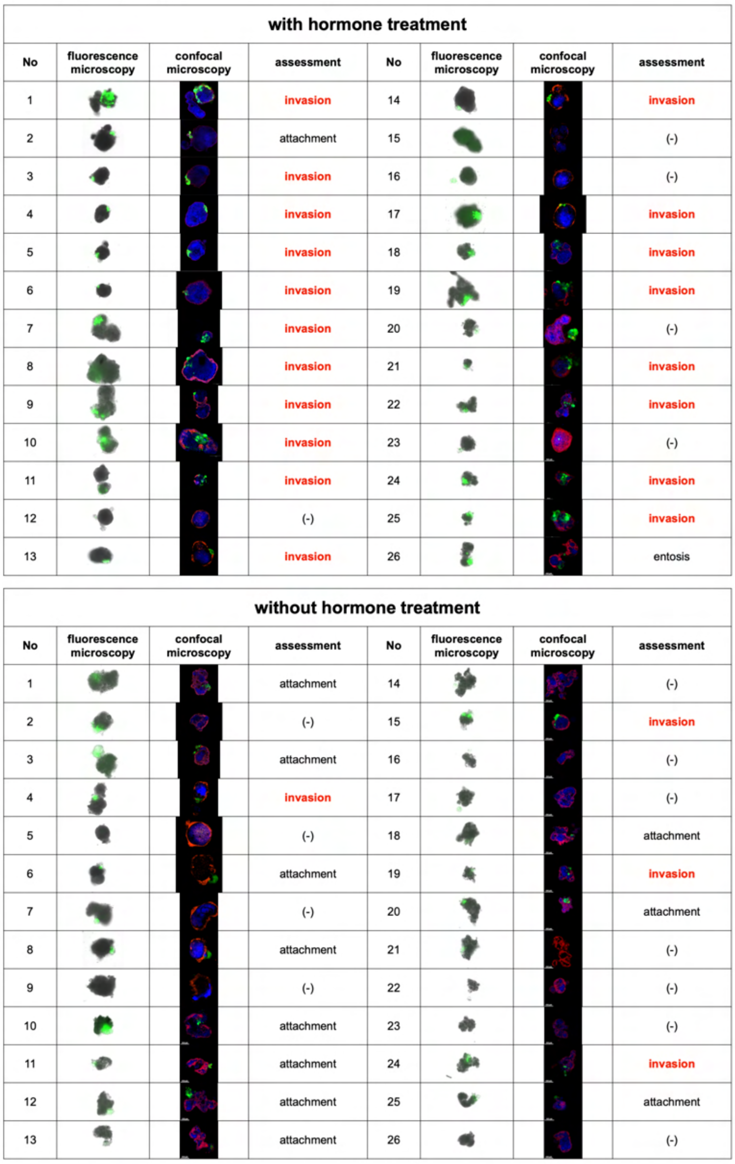

with hormone treatment

No	fluorescence microscopy	confocal microscopy	assessment	No	fluorescence microscopy	confocal microscopy	assessment
1			invasion	14			invasion
2			attachment	15			(-)
3			invasion	16			(-)
4			invasion	17			invasion
5			invasion	18			invasion
6			invasion	19			invasion
7			invasion	20			(-)
8			invasion	21			invasion
9			invasion	22			invasion
10			invasion	23			(-)
11			invasion	24			invasion
12			(-)	25			invasion
13			invasion	26			entosis

without hormone treatment

No	fluorescence microscopy	confocal microscopy	assessment	No	fluorescence microscopy	confocal microscopy	assessment
1			attachment	14			(-)
2			(-)	15			invasion
3			attachment	16			(-)
4			invasion	17			(-)
5			(-)	18			attachment
6			attachment	19			invasion
7			(-)	20			attachment
8			attachment	21			(-)
9			(-)	22			(-)
10			attachment	23			(-)
11			attachment	24			invasion
12			attachment	25			attachment
13			attachment	26			(-)

B

	Hormone treatment		
	(+)	(-)	p-value
Implantation rate	73.1% (19/26)	15.3% (4/26)	0.000059

Fig. S16. Effect of hormone pretreatment on in vitro implantation.

(A) The presence or absence of implantation were evaluated by whole organoid imaging in multiple samples (n=26 in each group). Bright filed images, whole organoid immunostaining images (RFP for E-cadherin, GFP for blastocyst-derived cell) in each sample are shown. The presence of implantation was defined as the invasion of GFP-positive blastocyst-derived cells within the ESC layer under EECs (shown in bold red). The degree of the implantation progress is shown in the right column.

(B) The implantation rate in the hormone-pretreated group was 73.1% (19/26), significantly higher than the 15.3% (4/26) in the untreated group (p<0.01, Fisher's exact test).

Development • Supplementary information

Table S1. Antibody details for Immunofluorescence labelling

Target	Host	Dilution	Supplier (Catalog.#)
Primary Antibodies			
E-Cadherin	Rabbit	1:200	Cell Signaling Technology (3195)
E-Cadherin	Mouse	1:50	Cell Signaling Technology (14472)
Vimentin	Rabbit	1:500	ABCAM (ab92547)
PR(Progesterone receptor)	Rabbit	1:100	ABCAM (ab101688)
MUC1	Rabbit	1:250	ABCAM (ab109185)
Proliferin	Mouse	1:200	SANTA CRUZ (sc-271891)
Cox2	Goat	1:50	SANTA CRUZ (sc-376861)
EpCAM	Rabbit	1:100	ABCAM (ab221552)
CK8	Mouse	1:50	SANTA CRUZ (sc-8020)
PDGFRα	Rabbit	1:1000	Cell Signaling Technology (3164)
FOXA2	Rabbit	1:300	ABCAM (ab108422)
FOXO1	Rabbit	1:500	SANTA CRUZ (sc-11350)
Secondary Antibodies			
Anti-Mouse, Alexa Fluor 488-conjugated	Goat	1:1000	ABCAM (ab150113)
Anti-Rabbit, Alexa Fluor 594-conjugated	Goat	1:1000	ABCAM (ab150084)
Anti-Goat, Alexa Fluor 647-conjugated	Donkey	1:1000	ABCAM (ab150131)
Anti-Mouse, Alexa Fluor 647-conjugated	Goat	1:1000	ABCAM (ab150115)

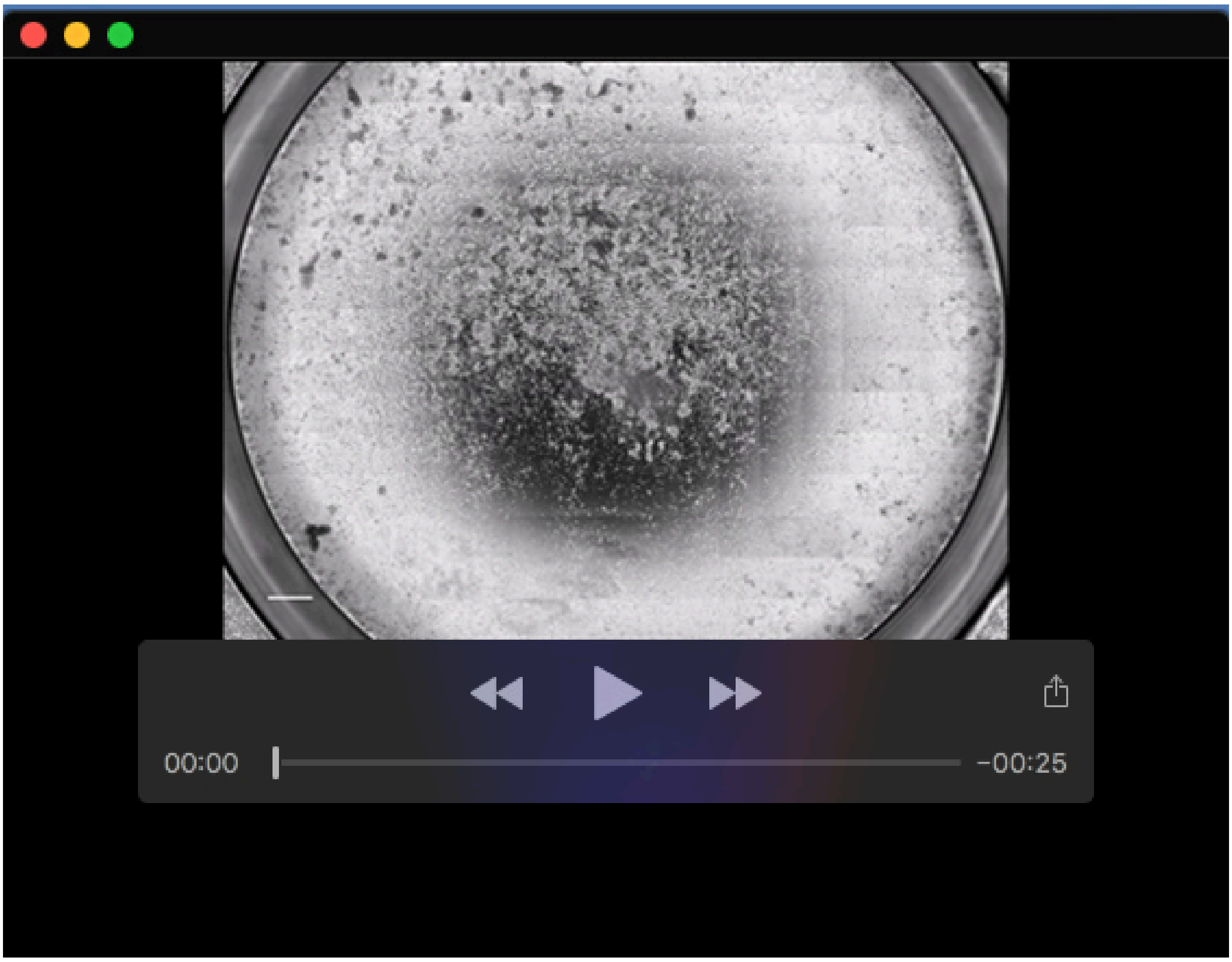

Movie 1. Time-lapse imaging of cell aggregation forming a large ring-like structure. The process of cell aggregation was monitored by time-lapse imaging from day 1 to day 3 after the initiation of adherent coculture. Scale bar, 1000 µm.

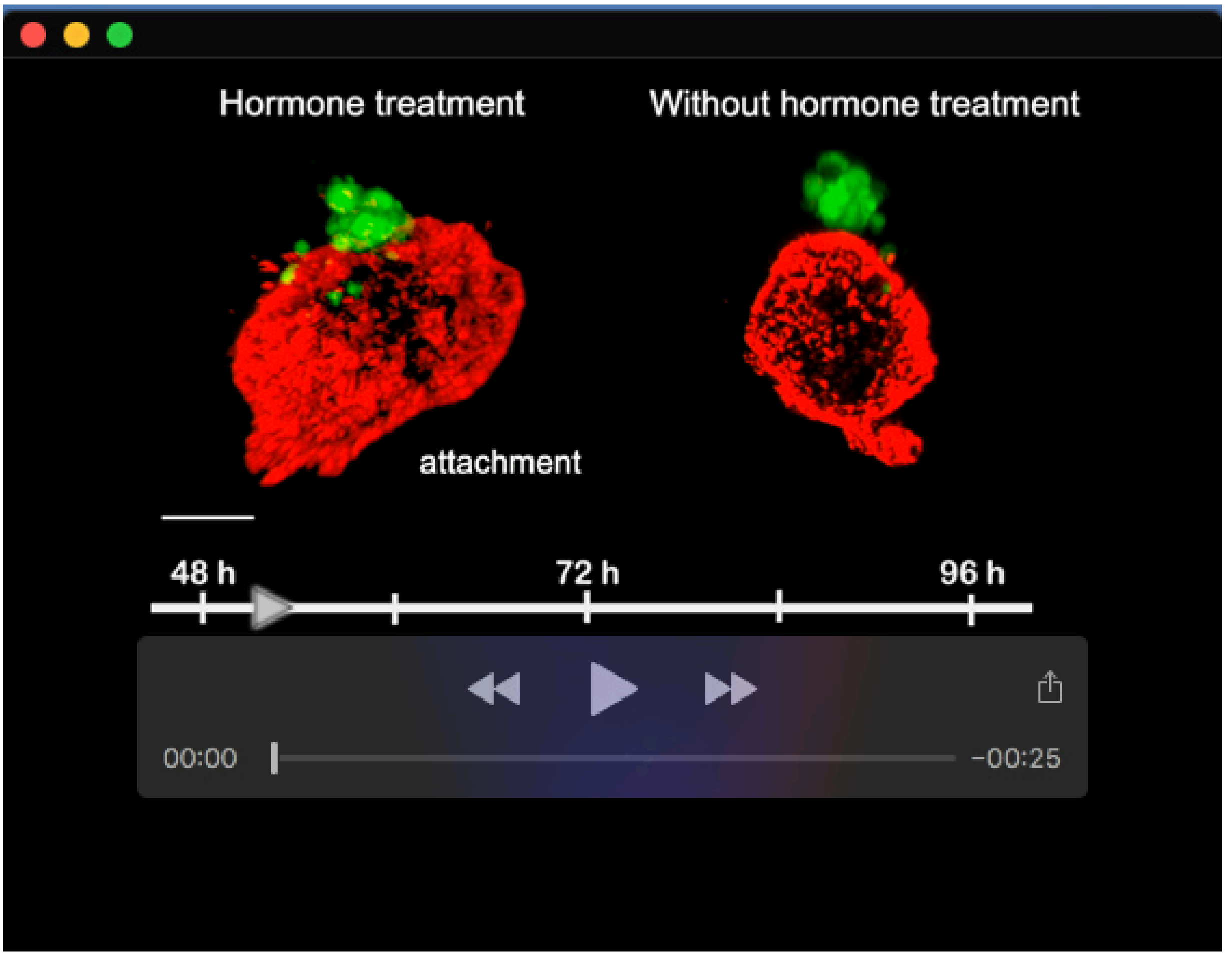

Movie 2. 3D time-lapse imaging of the in vitro implantation process.
The organoids were pretreated with or without hormones. Then, the organoids labeled with a red fluorescent were cocultured with blastocysts. The process of in vitro implantation was monitored using time-lapse imaging from 48 hours to 96 hours after the initiation of the co-culture. 3D images were reconstructed by Imaris. Scale bar, 50 µm.

Development • Supplementary information

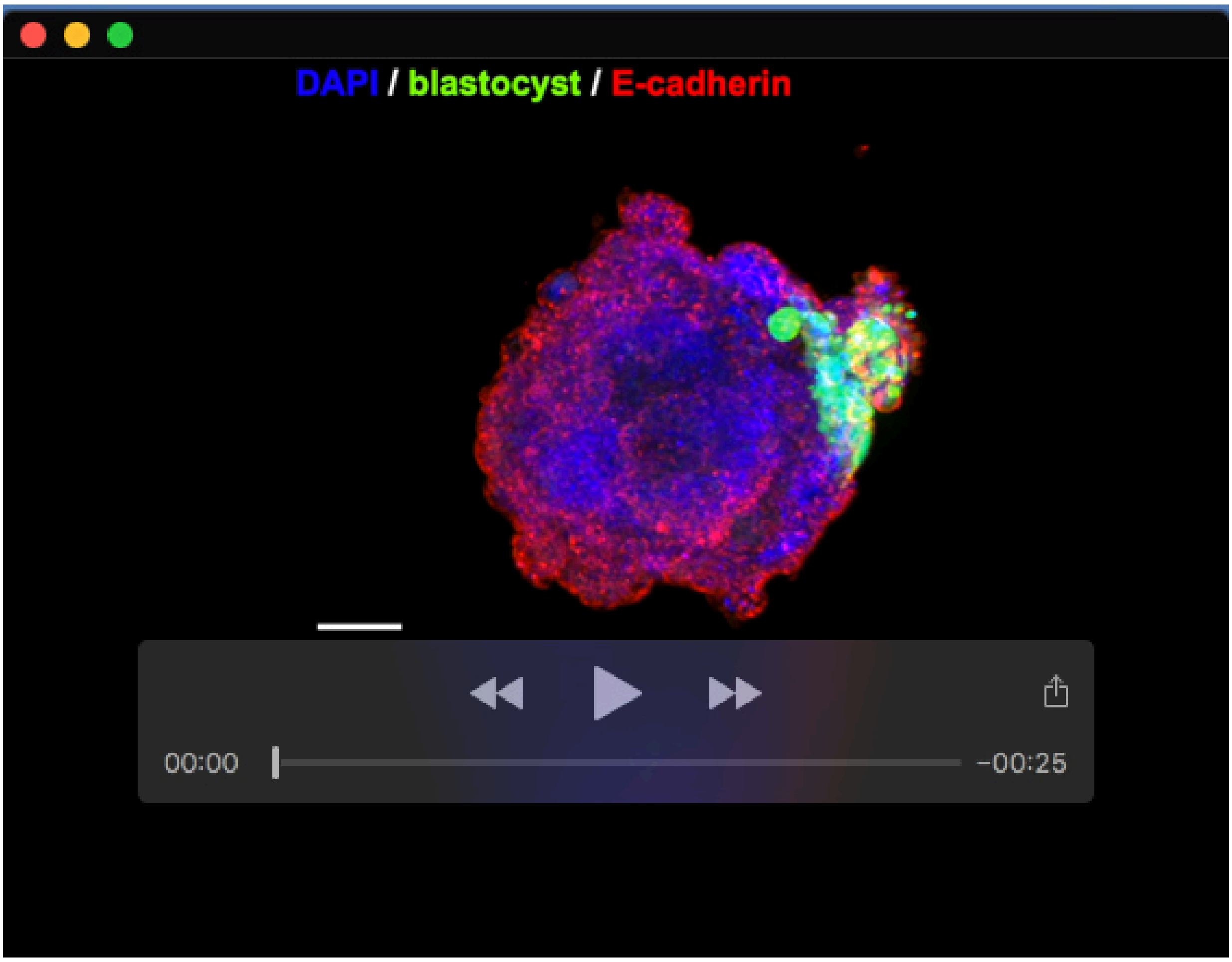

Movie 3. Representative whole organoid imaging of the implantation positive sample. After coculture with blastocysts, the organoids were cleared with a tissue-clearing reagent, and whole mount immunostaining for E-cadherin was performed to visualize EEC layer. The nuclei were stained with DAPI. GFP positive cells are blastocyst-derived cells. The images were taken by confocal microscopy and reconstructed in 3D. Scale bar, 100 µm.
